# Evaluation of a polysaccharide conjugate vaccine to reduce colonization by *Campylobacter jejuni* in broiler chickens

**DOI:** 10.1186/s13104-015-1203-z

**Published:** 2015-06-02

**Authors:** Douglas C Hodgins, Neda Barjesteh, Michael St. Paul, Zuchao Ma, Mario A Monteiro, Shayan Sharif

**Affiliations:** Department of Pathobiology, University of Guelph, Guelph, ON N1G 2W1 Canada; Department of Chemistry, University of Guelph, Guelph, Canada; Department of Immunology, University of Toronto, Toronto, ON Canada

**Keywords:** *Campylobacter jejuni*, Cecum, Capsular polysaccharide, Conjugate vaccine, Vaccination, Broiler chickens

## Abstract

**Background:**

*Campylobacter jejuni* is a leading bacterial cause of food-borne illness in humans. Symptoms range from mild gastroenteritis to dysentery. Contaminated chicken meat is the most common cause of infection. Broiler chickens become colonized with high numbers of *C. jejuni* in the intestinal tract, but do not become clinically ill. Vaccination of broiler chicks to control colonization by *C. jejuni* is challenging because immune function is limited in the first 2 weeks post-hatch and immune suppressive maternal antibodies are common. In addition, there is little time for induction of immunity, since broilers reach slaughter weight by 5–6 weeks of age. In the current study the immunogenicity of a *C. jejuni* capsular polysaccharide—diphtheria toxoid conjugated vaccine (CPSconj), administered subcutaneously with various adjuvants was assessed and the efficacy of vaccination for reducing cecal colonization after experimental challenge was evaluated by determining colony-forming units (CFU) of *C. jejuni* in cecal contents.

**Results:**

The CPSconj vaccine was immunogenic when administered as three doses at 3, 4 and 5 weeks of age to specific pathogen free chicks lacking maternal antibodies (seroconversion rates up to 75%). Commercial broiler chicks (having maternal antibodies) receiving two doses of CPSconj vaccine at 7 and 21 days of age did not seroconvert before oral challenge at 29 days, but 33% seroconverted post challenge; none of the placebo-injected, challenged birds seroconverted. Vaccinated birds had significantly lower numbers of *C. jejuni* in cecal contents than control birds at necropsy (38 days of age). CFU of *C. jejuni* did not differ significantly among groups of birds receiving CPSconj vaccine with different adjuvants. In two trials, the mean reduction in CFU associated with vaccination was 0.64 log_10_ units.

**Conclusions:**

The CPSconj vaccine was immunogenic in chicks lacking maternal antibodies, vaccinated beginning at 3 weeks of age. In commercial broiler birds (possessing maternal antibodies) vaccinated at 7 and 21 days of age, 33% of birds seroconverted by 9 days after challenge, and there was a modest, but significant, reduction in cecal counts of *C. jejuni*. Further studies are needed to optimize adjuvant, route of delivery and scheduling of administration of this vaccine.

## Background

Food-borne illness due to infection with *Campylobacter* species has been estimated to cost 1.7 billion dollars a year in medical costs, lost productivity and “quality-adjusted life years” in the United States alone [1]. Reports compiled by the European Food Safety Authority demonstrate increasing numbers of cases in humans over the most recent 4 years of study, in contrast to a steady decrease in the incidence of food-borne *Salmonella* infections [2]. Contaminated chicken meat is considered the most important source of infection with *Campylobacter* in developed countries [3]. Broiler chickens typically become infected with *Campylobacter jejuni* after 3 weeks of age and can harbor 10^8^ colony-forming units (CFU) or more per gram of cecal contents [4] by slaughter age (5–6 weeks of age). In contrast to the intense diarrhea and vomiting, and severe inflammation of intestinal tissues associated with *C. jejuni* infection in humans [5], chickens do not exhibit signs of clinical illness after colonization by *C. jejuni*, and inflammation of intestinal tissue is not evident histologically [6].

Enhanced biosecurity in poultry flocks and improved hygiene during processing of poultry products have potential to reduce contamination of meat at the retail level, but vaccination of broiler chickens will be needed in conjunction with these approaches to have a major impact on campylobacteriosis in humans [7]. At present there are no licensed vaccines for reduction of colonization of chickens by *C. jejuni* [8]. Various vaccine approaches have been explored in experimental studies in chickens (reviewed by de Zoete et al. [9]), including bacterins [10, 11], subunit vaccines [11], live *Salmonella*-vectored vaccines [12–14], and encapsulated particle vaccines [8, 15], by parenteral, oral, and nasal routes. Putative virulence factors and potential vaccine antigens have included outer membrane proteins [8], flagellin [11, 16], and transport proteins [12–14].

Recent studies have investigated the role of the capsular polysaccharide of *C. jejuni* in virulence in some species, and its potential as a vaccine antigen [17–20]. The capsular polysaccharide of *C. jejuni* 81-176 has been shown to mediate adherence and invasion of a human embryonic epithelial cell line, and to play a role in induction of diarrhea in a ferret model [21]. Wong et al. [22] have reported that modifications of the structure of the capsule of *C. jejuni* NCTC 11168 are associated with significant impairment of cecal colonization of young chicks. Capsular polysaccharide conjugated to the diphtheria toxoid “cross-reacting material 197” (CRM_197_) has been reported to be immunogenic in *Aotus* monkeys, and to protect against clinical diarrhea, but not colonization, following experimental challenge [17]. Although purified capsular polysaccharides can induce protection against encapsulated bacteria, as T-independent antigens they typically are not immunogenic in young infants or chicks [23, 24], and IgG and memory responses are limited [25]. Conjugation of purified capsular polysaccharide to a protein carrier such as CRM_197_ induces T-dependent responses, and facilitates antibody responses at an earlier age, with isotype switching to IgG and induction of B cell memory [26].

Although vaccination of broiler chicks is an attractive approach to control colonization, there are immunological and logistical barriers that must be overcome. Immune function is limited in the first 2 weeks post-hatch [27, 28] and maternal antibodies to *C. jejuni* are common in the sera of young chicks [29]. In addition there is little time for induction of immunity, since broiler birds reach slaughter weight by 5–6 weeks of age.

In the current studies the *immunogenicity* of the capsular polysaccharide of *C. jejuni* conjugated to CRM_197_ was assessed by vaccinating specific pathogen free (SPF) chicks (lacking maternal antibodies), starting at 3 weeks of age (when immune function has matured) and following serum antibody responses. In contrast, the *protective efficacy* of this antigen for reducing cecal colonization of broiler chicks was assessed by vaccinating commercial broiler chicks (having maternal antibodies) with two doses of vaccine (at 1 and 3 weeks of age), followed by experimental challenge at 4 weeks of age. Thus *immunogenicity* was tested under conditions favorable to induction of antibody responses, but *protective efficacy* was tested in commercial chicks using earlier vaccination, consistent with the need to induce immunity as young as possible.

## Methods

### Vaccine

Capsular polysaccharide of *C. jejuni* strain 81-176 was purified and conjugated to diphtheria toxoid CRM_197_ to form capsular polysaccharide conjugate (CPSconj) as described previously [17].

### Immunogenicity in the absence of maternal antibodies

Specific pathogen free chicks (SPF, N2 strain, Cornell University Center for Animal Resources and Education, Cornell, New York, USA, 5 chicks per experimental group), lacking detectable maternal antibodies at the time of vaccination, were raised in the isolation facilities of the Ontario Veterinary College and vaccinated subcutaneously at 3 and 4 weeks of age with 10 μg CPSconj with either 20 μg Quil A (Brenntag Biosector, Frederickssund, Denmark) or 10 μg CpG oligodeoxynucleotide (CpG ODN) 2007 (Eurofins Operon, Huntsville, AL, USA) as adjuvants, or received 20 μg CPSconj with 20 μg Quil A at 3, 4 and 5 weeks of age. Vaccination of these chicks thus occurred under favorable conditions (absence of maternal antibodies) beginning at an age when immune function is considered to be mature [27, 28]. Control (placebo) birds received phosphate buffered saline (PBS) in place of vaccine.

### Vaccination and challenge of commercial broiler chicks

One-day-old female commercial broiler chicks (Ross 308 chicks from Stratford Chick Hatchery, Stratford, ON, Canada) were housed in isolation facilities at the Ontario Veterinary College. Chicks were allocated (8 chicks per group) to receive 25 μg CPSconj with 10 μg CpG or 100 μl Addavax™ (squalene based adjuvant, InvivoGen, San Diego, CA, USA) as adjuvant, or 25 μg CPSconj without adjuvant, or PBS as a placebo by subcutaneous injection in a 200 μl volume at 7 days post-hatch. Birds received a booster dose of 10 μg of the same antigen preparation as previously, or PBS at 21 days post-hatch. In contrast to the chicks used in the immunogenicity experiment above, these broiler chicks were obtained from a commercial source and had detectable serum (maternal) antibodies at the time of primary vaccination. At 29 days post-hatch birds were challenged orally with 2 × 10^7^ CFU of *C. jejuni* strain 81-176 prepared by the method of Davis and DiRita [30] (see below). Birds were necropsied at 9 days post-challenge (38 days post-hatch) and dilutions of the cecal contents were plated onto Mueller–Hinton agar containing Preston Campylobacter Selective Supplement (Oxoid, Basingstoke, Hampshire, UK) and incubated at 42°C for 48 h in a microaerobic environment to quantitate CFU of *C. jejuni*. CFU per gram of cecal contents were calculated based on plate counts, adjusting for dilutions. An additional eight birds (serving as negative controls for challenge) were housed in a separate isolation room, were not vaccinated and were not challenged, but were necropsied at the same time as challenged birds. Blood was collected at 7, 21, 29, 34 and 38 days post-hatch for serological testing. The experiment was repeated a second time using chicks from the same source, following the identical protocol in the same facilities, and the results were pooled for statistical analysis.

### Animal ethics

All experiments were approved by the Animal Care Committee of the University of Guelph (Animal Utilization Protocol number 10R086-1836) and followed the guidelines of the Canadian Council on Animal Care.

### Enzyme-linked immunosorbent assay (ELISA) for serum IgG antibodies

Purified capsular polysaccharide of *C. jejuni* diluted to 40 μg/ml in PBS buffer was coated onto Nunc 96 well (MicroWell™ untreated polystyrene, Thermo Fisher Scientific, Rochester, New York, USA) plates, 100 μl/well, at 37°C for 3 h. Plates were washed four times with wash buffer consisting of PBS with 0.5% fish skin gelatin (Sigma, St. Louis, MO, USA) and 0.05% Tween 20 (Sigma). Sera were diluted 1/20 in wash buffer and 100 μl was added to wells in duplicate, followed by a 2 h incubation at 37°C. After washing four times, bound antibodies were detected using rabbit anti-chicken IgG (Fc specific) horse radish peroxidase conjugate (Jackson ImmunoResearch Laboratories, West Grove, PA, USA) diluted 1/350, incubating at 37°C for 1 h. After washing four times, ABTS (2,2′-azino-di (3-ethyl-benzthiazoline-6-sulfonate)) substrate (Kirkegaard and Perry Laboratories, Gaithersburg, Maryland, USA) was added, 100 μl/well; 1% sodium dodecylsulfate (Bio-Rad, Hercules, CA, USA) was added as stop solution after 1 h and the optical densities were evaluated at 405 nm. A dilution series of a positive control serum consisting of pooled high titre sera from mature hens was run on each plate. Titres were estimated using the method of Sacks et al. [31]. The limit of detection of the assay was 3.32 log_2_ units.

### Bacterial culture

*Campylobacter jejuni* strain 81-176 was cultured according to the method of Davis and DiRita [30] to provide bacteria for experimental oral challenge. Briefly, a 10-μL loop of frozen *C. jejuni* culture maintained at −80°C, was inoculated onto Mueller–Hinton agar (Oxoid) and incubated for 18 h in a sealed jar using gas packs (CampyGen, Oxoid) to maintain microaerobic conditions. Subsequently, several colonies of *C. jejuni* were inoculated into 100 mL fresh Mueller–Hinton broth and incubated at 41°C in microaerobic conditions for 40 h. The broth culture was centrifuged at 3,500×*g* for 10 min and the bacteria were diluted with PBS (pH 7.4) to attain an optical density corresponding to approximately 4.0 × 10^7^ CFU/ml, based on previous analysis of growth curves. The number of viable *C. jejuni* received by the chickens at the time of challenge was established retrospectively by plating dilutions of the inocula onto Mueller–Hinton agar plates.

### Statistical analysis

Data from the two challenge trials were pooled. CFU/gm of *C. jejuni* in cecal contents were log_10_ transformed before analysis using a mixed model (Proc Mixed) including trial as a random variable, in SAS version 9.2. Vaccine groups were subsequently compared using Tukey’s test. Antibody titres were expressed on a log_2_ scale. Titres were not normally distributed for the majority of time points; a nonparametric test (Proc Npar1way [Kruskal–Wallis test]) was used to compare antibody titres among groups. Seroconversion (increase in titre by ≥2 log_2_ units) rates were compared using Fisher’s exact test. Correlation between serum antibody titres on the day of necropsy and CFU of *C. jejuni* in cecal contents was evaluated using Pearson’s correlation coefficient using Proc Corr in SAS.

## Results and discussion

### Immunogenicity of the CPS conjugate vaccine in the absence of maternal antibodies

**Figure 1:**
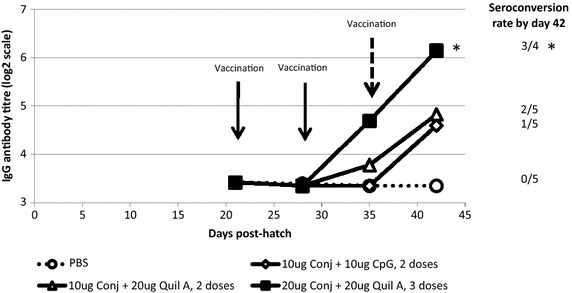
Immunogenicity of the capsular polysaccharide-diphtheria toxoid (CRM_197_) conjugate vaccine. Specific pathogen free chicks (N2 strain), lacking maternal antibodies at the time of primary vaccination, were vaccinated subcutaneously at 3 and 4 weeks post-hatch (10 μg of conjugate with 10 μg CpG or with 20μg Quil A), or at 3, 4 and 5 weeks post-hatch (20 μg of conjugate with 20 μg Quil A) or received phosphate buffered saline (PBS) as a placebo. Mean serum IgG antibodies specific for capsular polysaccharide of *C. jejuni* strain 81-176 were assayed by ELISA and titres were expressed on a log_2_ scale. The limit of detection was 3.32 (log_2_ scale). Seroconversion [fourfold or greater increase in antibody titre (≥2 log_2_ units)] rates after vaccination are indicated on the right of the figure for each group. * Seroconversion rate was significantly higher than in birds receiving PBS as placebo (p < 0.05, one-tailed Fisher’s exact test).

Mean serum IgG antibody titres increased in vaccinated SPF birds, but not in negative control birds that received PBS (Figure 1). Seroconversion rates were 20, 40 and 75% by 6 weeks of age in birds receiving two doses of CPSconj vaccine with CpG, two doses CPSconj with Quil A and three doses CPSconj with Quil A respectively. The seroconversion rate in birds receiving three doses of CPSconj was significantly higher than the rate in birds receiving PBS as placebo (p < 0.05, one-tailed Fisher’s exact test).

### Antibody responses of commercial broiler chicks

**Figure 2:**
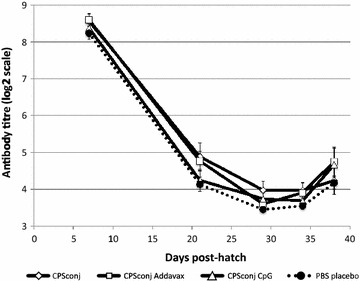
Mean serum IgG antibody titres to capsular polysaccharide of *C. jejuni* strain 81-176. Serum antibody titres were determined by ELISA. Error bars represent the standard error of the mean. At no point in time did the titres differ significantly among the experimental groups (Kruskal–Wallis nonparametric test). By the day of necropsy (day 38, 9 days post-challenge) 33% of vaccinated birds and 0% of PBS placebo birds had seroconverted with a four-fold or greater increase in antibody titre (≥2 log_2_ units), (see Figure 3).

Maternal IgG antibodies were evident in serum at 7 days post-hatch at the time of primary vaccination, but declined considerably by day 21 (day of secondary vaccination, Figure 2). Maternal antibodies have been shown to mediate partial protection against colonization by *C. jejuni* [32], but the short half-life of maternal antibodies (about 5 days in young chicks [33]) suggests that titres of maternal antibodies would need to be considerably higher to provide protection up to slaughter age. Mean serum IgG antibody titres declined further by day of challenge (day 29) in all groups. IgG antibody titres did not differ significantly among the four experimental groups (three groups of vaccinates and one placebo group) at any of the time points (Kruskal–Wallis test). Data from the vaccinated groups (CPS conjugate without adjuvant, CPS conjugate with Addavax™ adjuvant and CPS conjugate with CpG as adjuvant) were pooled for further analysis since there were no significant differences among them.

**Figure 3:**
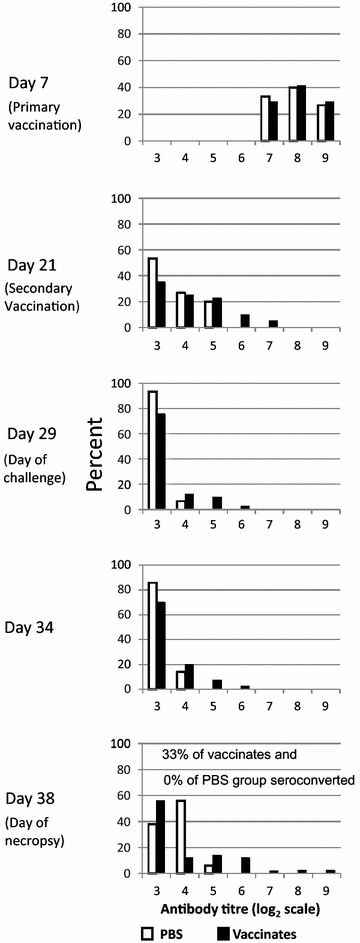
Histograms showing distribution of serum IgG antibodies specific for capsular polysaccharide of *C. jejuni* strain 81-176 in commercial broiler chicks. Chicks were vaccinated at 7 and 21 days post-hatch with capsular polysaccharide-conjugate (with CpG ODN 2007, or Addavax™ as adjuvant, or no adjuvant; antibody titres for the three vaccinated groups were pooled to graph the histograms since they did not differ significantly at any of the time points) or received PBS placebo. IgG antibody titres were determined by ELISA and analyzed using the Kruskal–Wallis (nonparametric) test because at the majority of time points, the titres were not normally distributed. In each panel, the percentage (Y-axis) of (vaccinated and PBS control) birds with particular antibody titres is plotted against their titres at that time point. A shift (to the *left*) in the distribution of antibody titres, consistent with decline in titres of maternal antibodies, is evident from day 7 post-hatch to day 29 (day of challenge), followed by a shift to the *right* after challenge. Antibody titres did not differ significantly among the four treatment groups at any of the time points. Seroconversion, a fourfold or greater (≥2 log_2_ units) increase in antibody titre, did not occur in any of the birds before the day of challenge (day 29 post-hatch), but 33% of vaccinated and 0% of placebo (PBS) birds seroconverted by day 38 (9 days post-challenge, a significant difference, Fisher’s exact test p < 0.01). For each time point, sera from 14 to 16 PBS (placebo) birds and sera from 40 to 43 vaccinated birds were assayed.

In Figure 3 the distribution of serum IgG antibody titres in vaccinated broiler birds and birds receiving placebo, is contrasted by means of histograms. Seroconversion (an increase of titre ≥2 log_2_ units) was not detected before day of challenge in vaccinates. This is in contrast to responses seen in SPF birds by 3 weeks post primary vaccination (Figure 1). Seroconversion occurred in 33% of vaccinated broiler birds by 9 days post-challenge, but in none of the birds that had received placebo before challenge (p < 0.01, Fisher’s exact two-tailed test). This suggests that although vaccination with two doses of vaccine did not induce increases in circulating antibody titres before the day of challenge, there was a degree of priming for anamnestic antibody responses (which occurred after exposure to live *C. jejuni*) in some of the birds. In a similar fashion, Siegrist et al. [34] have reported an absence of serum antibody responses to vaccination in neonatal mice with circulating maternal antibodies. In these mouse experiments anamnestic antibody responses became evident after subsequent antigen exposure.

It is possible that if cecal CFU were monitored beyond 9 days post-challenge, that mean CFU would decline further as immune responses aided in clearance of *C. jejuni*. A gradual decline in cecal colonization in birds vaccinated with live attenuated *Salmonella* expressing the CjaA protein of *C. jejuni* has been reported by Buckley et al. [14]. Any decline after 5–6 weeks of age, however, is not relevant since most broiler birds are slaughtered by that age. Clearly further research will be needed to improve the immunogenicity of the vaccine for young broilers.

The lower seroconversion rates noted in commercial broiler chicks compared to SPF N strain birds are most probably due to the younger age at primary vaccination of the broilers, or to suppressive effects of residual maternal antibodies, but the effect of different genetic backgrounds cannot be eliminated as a factor in the current study.

## Reduction in *C. jejuni* in cecal contents of vaccinated chicks

Colony-forming units of *C. jejuni* were significantly lower in the cecal contents of birds receiving CPSconj vaccine (with or without adjuvant) compared to control birds receiving PBS (Table 1). Mean CFU of *C. jejuni* did not differ significantly among groups of vaccinated birds and were pooled for further analysis. Vaccinated birds had a mean reduction in cecal *C. jejuni* of 0.64 log_10_ units compared to birds receiving PBS as placebo (p < 0.001). There was essentially no correlation between serum antibody titres and cecal counts of *C. jejuni* at necropsy (r value of −0.06 among vaccinates with p = 0.70, data not shown). Since *Campylobacter* colonize the mucus layer of the intestinal tract and are rarely found in extra-intestinal sites, a low correlation between serum antibody titres and protection could be anticipated. Assay of antibodies in intestinal mucus should be more informative. Vaccination with CPSconj by a mucosal route might improve vaccine efficacy compared to the subcutaneous route used in the present study, by inducing IgA antibodies at sites of colonization by *C. jejuni*. The subcutaneous route was used in the present study to assess immunogenicity of the vaccine construct and to provide a baseline for comparison as systems for delivery to mucosal sites are developed. Rosenquist et al. [35] have suggested, based on mathematical modeling, that a 2.0 log_10_ unit reduction in *C. jejuni* in chickens at slaughter would reduce clinical illness in humans 30-fold. Buckley et al. [14] have reported reductions in cecal colonization of 1.57–1.91 log_10_ CFU of *C. jejuni* per gram of cecal contents in SPF chickens (lacking maternal antibodies) vaccinated with recombinant CjaA protein, but reductions were not evident until 7 weeks of age. Widders et al. [11] have reported reductions of 1.9 log_10_ CFU per gram of cecal contents by 6 weeks of age in birds vaccinated intraperitoneally with antigens of killed *C. jejuni*. Larger reductions in cecal counts of *C. jejuni* (6 log_10_ CFU per gram) have been reported with *Salmonella* vectored vaccines [12], but there are safety issues associated with persistence of the *Salmonella* vector in vaccinated birds [14].

**Table 1:** Colony-forming units of *C. jejuni* per gram of cecal contents 9 days post-challenge

Vaccine group	n	CFU per gram of cecal contents (log_10_ transformed)	Standard error of the mean	p value for comparison with the PBS group
PBS	16	8.11	0.15	
Conjugate alone	13	7.38	0.16	0.01
Conjugate + CpG	14	7.55	0.15	<0.05
Conjugate + Addavax™	16	7.47	0.14	0.01
All vaccinates (data pooled)	43	7.47	0.09	<0.001

Combined results from two experiments using Ross 308 broiler chicks from the same source and following the same experimental protocol. Chickens were vaccinated subcutaneously at 7 and 21 days post-hatch with capsular polysaccharide conjugate vaccine with or without CpG or Addavax™ as adjuvant, or received PBS as a placebo. Birds were challenged at 29 days post-hatch and necropsied at 38 days post-hatch.

A previous study vaccinating mature monkeys (lacking pre-existing antibody titres) with a CPSconj vaccine has demonstrated efficacy in reducing clinical signs (in primates), but quantitative studies of intestinal carriage of *C. jejuni* were not carried out [17]. Because *C. jejuni* causes clinical disease in primates, the end goal of vaccination in such studies is reduction in signs of disease. In contrast the goal in vaccinating chickens is to reduce the number of *C. jejuni* in the intestinal tract of the birds at the time of slaughter. This may require a qualitatively different or quantitatively greater immune response and will be a more elusive goal.

## Alternative vaccination approaches

The current study assessed immunogenicity of the capsular polysaccharide antigen conjugated to diphtheria toxoid CRM_197_. This carrier protein has been used in vaccines for human infants and has been shown to enhance immune responses to polysaccharide antigens in the first months of life [26]. Other carrier proteins have been used in conjugate vaccines [36] and perhaps these or even *C. jejuni* surface proteins may also prove efficacious in chickens. Schijns et al. [37] have reported that some adjuvants enhance the immunogenicity of tetanus toxoid in immunologically mature chickens (3 weeks-old) but fail to induce antibodies to this antigen in day-old chicks, whereas other adjuvants are effective at both ages. Thus it may be necessary to optimize both carrier protein and adjuvant to produce conjugate vaccines immunogenic for day-old chicks.

In the present study vaccines were administered subcutaneously to evaluate immunogenicity of the capsular antigen. Mucosal vaccination, especially by the oral route may be more effective to induce intestinal immune responses and reduce intestinal colonization, but improved adjuvants and delivery systems will be required to make this approach practical.

Further studies will be needed to optimize adjuvants, route of delivery, and scheduling of administration for use of CPSconj antigen in broiler chickens. Studies using heterologous challenge strains of *C. jejuni* are also in order.

## Conclusions

A capsular polysaccharide—diphtheria toxoid conjugate vaccine administered subcutaneously, starting at 3 weeks of age, induced serum IgG antibodies in SPF chicks lacking detectable maternal antibodies. Vaccination of broiler chicks (that had maternal antibodies) starting at 7 days post-hatch was not associated with seroconversion with IgG antibodies before challenge at 29 days post-hatch. Some (33%) of the vaccinated broiler birds seroconverted by 9 days post-challenge, but none of the PBS (placebo) birds, indicating that the vaccine primed for an earlier IgG antibody response following challenge. There was a modest (0.64 log_10_ colony forming units) but significant (p < 0.001) reduction in *C. jejuni* in cecal contents, 9 days post-challenge, in vaccinated birds compared to negative control (placebo) birds. Further studies are needed to optimize adjuvant, route of delivery and scheduling of administration to make the most effective use of this vaccine antigen.

## Abbreviations

CFU: colony forming units
PBS: phosphate buffered saline
ODN: oligonucleotide
CPSconj: capsular polysaccharide-diphtheria toxoid conjugate
SPF: specific pathogen free
